# Genomic loss of heterozygosity and survival in the REAL3 trial

**DOI:** 10.18632/oncotarget.26336

**Published:** 2018-11-30

**Authors:** Elizabeth C. Smyth, Catherine Cafferkey, Andrea Loehr, Tom Waddell, Ruwaida Begum, Clare Peckitt, Thomas C. Harding, Minh Nguyen, Alicia F. Okines, Mitch Raponi, Sheela Rao, David Watkins, Naureen Starling, Gary W. Middleton, Jonathan Wadsley, Wasat Mansoor, Tom Crosby, Andrew Wotherspoon, Ian Chau, David Cunningham

**Affiliations:** ^1^ Department of Gastrointestinal Oncology and Lymphoma, Royal Marsden Hospital, London & Sutton, United Kingdom; ^2^ Current affiliation: Cambridge University Hospitals NHS Foundation Trust, Cambridge, United Kingdom; ^3^ Clovis Oncology, San Francisco, CA, United States of America; ^4^ Current affiliation: Department of Medical Oncology, Christie Hospital, Manchester, United Kingdom; ^5^ Department of Clinical Research & Development, Royal Marsden Hospital, London & Sutton, United Kingdom; ^6^ Institute of Immunology and Immunotherapy, University of Birmingham, Birmingham, United Kingdom; ^7^ Department of Medical Oncology, Weston Park Hospital, Sheffield, United Kingdom; ^8^ Department of Clinical Oncology, Velindre Hospital, Cardiff, Wales, United Kingdom; ^9^ Department of Histopathology, Royal Marsden Hospital, London & Surrey, United Kingdom

**Keywords:** gastric cancer, oesophageal cancer, chemotherapy, homologous recombination deficiency, loss of heterozygosity

## Abstract

**Background:**

Homologous recombination deficiency (HRD) measured using a genomic signature for loss of heterozygosity (LOH) predicts benefit from rucaparib in ovarian cancer. We hypothesized that some oesophagogastric cancers will have high-LOH which would be prognostic in patients treated with platinum chemotherapy.

**Methods:**

Diagnostic biopsy DNA from patients treated in the REAL3 trial was sequenced using the Foundation Medicine T5 next-generation sequencing (NGS) assay. An algorithm quantified the percentage of interrogable genome with LOH. Multidimensional optimization was performed to identify a cut-off dichotomizing the population into LOH-high and low groups associated with differential survival outcomes.

**Results:**

Of 158 available samples, 117 were successfully sequenced; LOH was derived for 74 of these. A cut-off of 21% genomic LOH defined an LOH-high subgroup (n=10, 14% of population) who had median overall survival (OS) of 18.3 months (m) versus 11m for the LOH-low group (HR 0.55 95% CI 0.19-0.97, p= 0.10). Progression free survival (PFS) for LOH-high and LOH-low groups was 10.7m and 7.3m (HR 0.61 (95% CI 0.21 – 1.09, p=0.09). Sensitivity analysis censoring operated patients (n=4), demonstrated OS of 18.3m vs. 10.2m (HR 0.43, 95% CI (0.20-0.92), p=0.02; PFS was 10.5m vs. 7.2m (HR 0.55, (95% CI 0.26-1.17), p=0.09 for LOH-high and LOH-low.

**Conclusion:**

HRD assessment using an algorithmically derived LOH signature on a standard NGS panel identifies oesophagogastric cancer patients with high LOH who have prolonged survival when treated with platinum chemotherapy. Validation work will determine the signature's predictive value in patients treated with a PARP inhibitor and with platinum chemotherapy.

## INTRODUCTION

Homologous recombination (HR) is a complex process requiring the coordinated function of a number of genes products in order to repair double-stranded breaks in DNA [1]. This process is frequently deranged in cancer, where the classic example of homologous recombination deficiency (HRD) is provided by BRCA1/2 mutated tumours [2, 3]. Targeting HRD in *BRCA* mutant tumours using a synthetically lethal approach with poly ADP ribose polymerase (PARP) inhibitors has resulted in beneficial effects for patients in ovarian, breast and prostate cancer [4–7]. However, HRD may also be present in tumours without BRCA mutations; similar to BRCA mutant tumours these cancers are often platinum sensitive and may also respond to other DNA damage targeting drugs [7–10]. Such *BRCA* wild-type HRD tumours have high levels of “genomic scarring”, which arises from the use of error prone DNA repair pathways when homologous recombination is compromised. One method for quantifying the amount of genomic scarring is to assess the extent of loss of heterozygosity (LOH; loss of one copy of a chromosomal region) across the tumour genome. Determination of HRD using LOH may have clinical implications; in the ARIEL2 Part 1 (NCT01891344) trial of previously treated ovarian cancer, *BRCA* wild-type patients with high levels of LOH (LOH-high) treated with the PARP inhibitor rucaparib were more likely to respond to rucaparib therapy and had longer progression free survival compared to rucaparib-treated patients who were not LOH-high [7, 11].

Oesophagogastric cancer is a platinum sensitive disease in which several genomic and proteomic biomarkers associated with DNA repair defects have been identified. These include ATM loss, The Cancer Genome Atlas (TCGA) chromosomally unstable (CIN) subtype and a putative BRCA mutational signature [12–16]. Therefore we hypothesized that genomic LOH (as a measure of HRD) might be associated with prognosis in oesophagogastric cancer patients treated with platinum based chemotherapy. In order to examine this hypothesis we assessed genomic LOH in tumour samples from patients treated with epirubicin, oxaliplatin and capecitabine plus or minus panitumumab (EOX±P) in the REAL3 (Randomised Trial of EOX with or without Panitumumab in Advanced or Locally Advanced Oesophagogastric Cancer 3) Trial, (NCT00824785), and correlated LOH with survival in this patient cohort.

Details of the REAL3 trial have been previously described [17]. In brief, eligible patients had a diagnosis of locally advanced or metastatic oesophagogastric cancer and were treated with EOX (epirubicin, oxaliplatin and capecitabine) plus or minus panitumumab (a fully human monoclonal IgG2 anti-EGFR antibody). Patients treated with EOX-panitumumab had inferior overall survival compared with patients treated with EOX (HR 1.37, 95% CI 1.07-1.76; p=0.013).

## RESULTS

Out of a total of 553 REAL3 patients 158 formalin-fixed paraffin embedded (FFPE) tissue blocks with high tumour content (>30%) were available; these were sent for NGS analysis to Foundation Medicine (FM). There was no significant difference in clinicopathological characteristics, progression free survival or overall survival between patients who underwent sequencing for LOH assessment and those without (see Supplementary Data). Of the 158 samples, one duplicate sample from the same patient was excluded. This left 157 samples which were processed for NGS (see CONSORT figure, Supplementary Data). The sample storage time for selected archival samples was a median of 5 years (range 4-9 years) and the quality of FFPE tissue blocks varied widely across the processed batch. Following review of tumor nuclei enumeration at FM, 5 samples were deemed to have too low tumour content and were excluded from further analysis. We observed attrition during NGS processing due to tissue quality in 35 samples (23%) and were able to sequence 117 samples successfully. The LOH inference was successfully performed for 74 of the sequenced samples (63%) or 47% of the original biomarker analysis population. This is because inference of LOH is based on copy number estimation which requires adequately deep and relatively even coverage across the genome. In contrast to mutation calling, the sample quality requirements for copy number detection are higher. Since the REAL3 samples had not been fixed with NGS in mind, not all samples met the higher quality standards for LOH inference but were sufficient for mutation calling.

There was no difference in the proportion of samples which were successfully sequenced from each tumour site; however junctional and oesophageal tumours were more likely to have LOH successfully inferred than stomach cancer (73% and 66% vs 50% respectively). Table 1 summarizes the number of samples sequenced and the proportion of samples which had LOH derived according to anatomical site.

**Table 1:** Number of samples sequenced and LOH derived by anatomical site

	Overall	Stomach	Oesophagus	GOJ
**Samples submitted**	157	46	56	55
**Samples sequenced**	117 (75%)	36 (78%)	41 (73%)	40 (73%)
**Samples with LOH derived**	74 (47%)	18 (50%)	27 (66%)	29 (73%)

**Figure 1:**
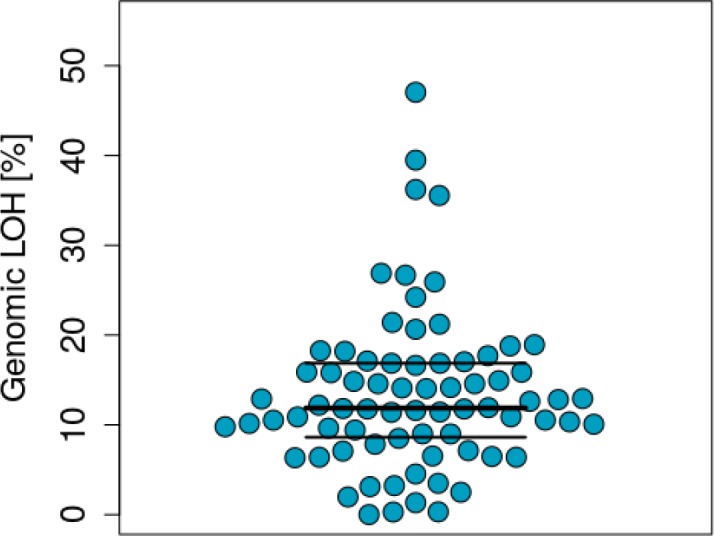
Distribution of genomic LOH across the samples analysed

**Figure 2:**
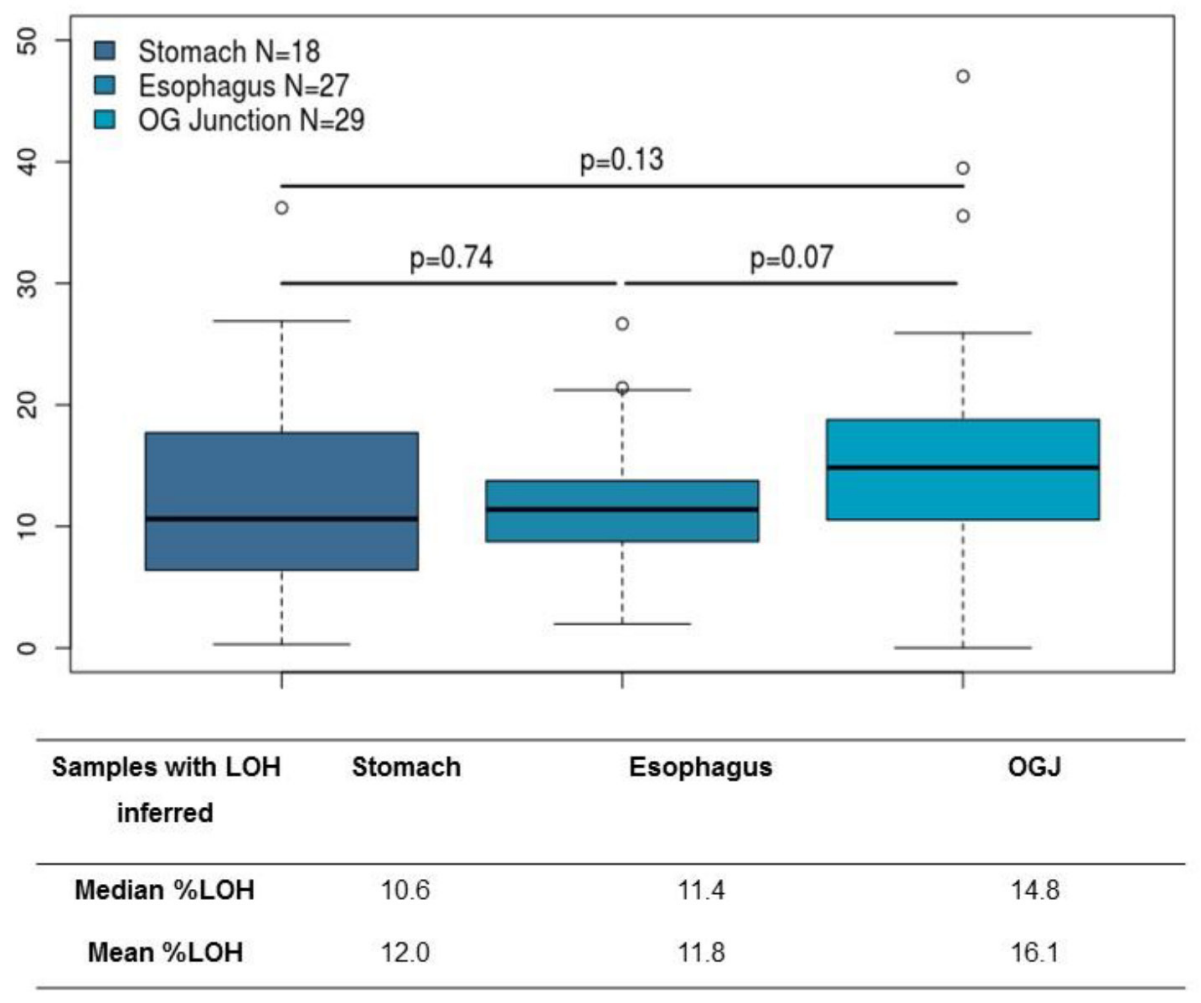
Genomic loss of heterozygosity by tumour site

The median percentage of genomic LOH inferred for all tumours was 11.9% (n=74) (Figure 1). According to anatomical site, the median percentage of LOH was 10.6% for stomach (n=18), 11.4% oesophagus (n=27) and 14.8% (n=29) for gastroesophageal junction tumours. These differences were not statistically significant (p>0.05). The median and mean % LOH according to anatomical site are shown in Figure 2.

**Figure 3:**
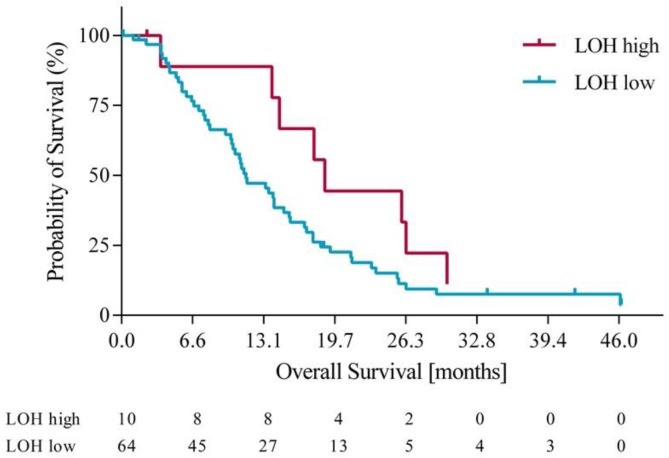
Overall survival in LOH-high and LOH-low groups

**Figure 4:**
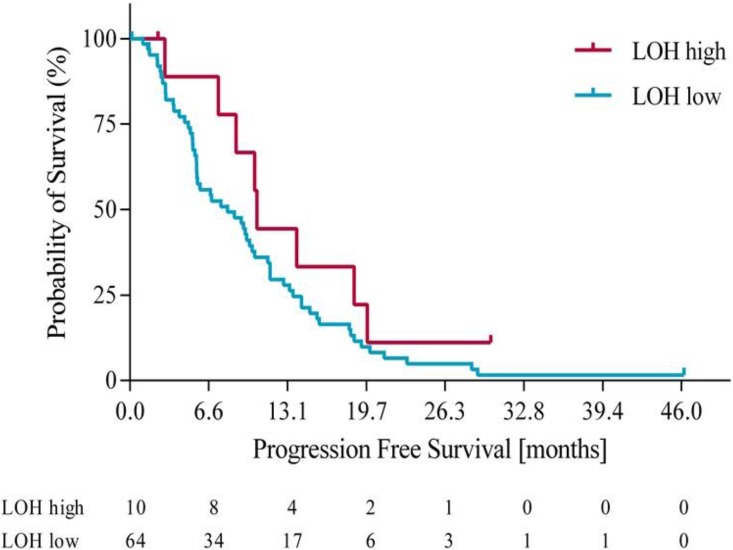
Progression free survival in LOH-high and LOH-low groups

**Figure 5:**
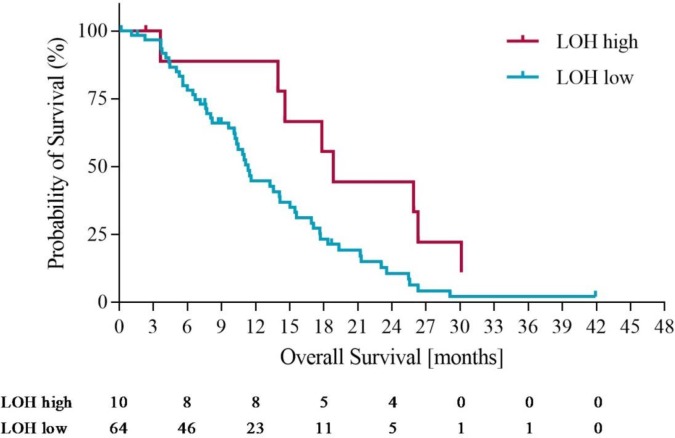
Overall survival in LOH-high and LOH-low groups with operated patients censored at time of potentially curative surgery

**Figure 6:**
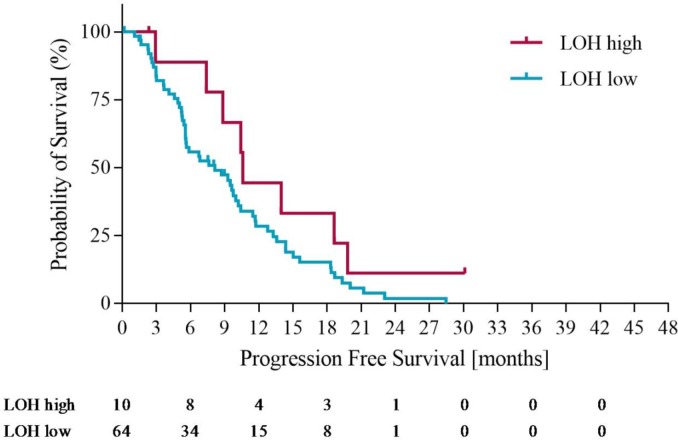
Progression free survival in LOH-high and LOH-low groups with operated patients censored at time of potentially curative surgery

Using the optimization of survival benefit as described in the methods section, the optimal survival benefit for LOH-high vs. LOH-low patients was found to be in patients with ≥ 21% genomic LOH. Using an LOH level of ≥ 21% to define the LOH-high group, 10 out of 74 patients (14%) were classified as LOH-high. At this cut off, the median overall survival (OS) was 18.3 months for the LOH-high subgroup compared to 11 months for the LOH-low subgroup. Using a Cox proportional hazards model, we derived the OS hazard ratio to be 0.55 (95% CI 0.19-0.97), p= 0.10. At the same LOH cut off, progression free survival (PFS) was 10.7 months for the LOH-high group compared to 7.3 months for the LOH-low group. Using a Cox proportional hazards model, the PFS HR was 0.61 (95% CI 0.21 – 1.09), p=0.09. Figures 3 and 4 illustrate the overall survival and progression free survival curves in LOH-high and LOH-low subgroups. In the sensitivity analysis in which operated patients (n=4) were censored at the time of potentially curative surgery OS of 18.3m vs. 10.2m (HR 0.43, 95% CI (0.20-0.92), p=0.02) for LOH-high vs. LOH-low patients and and PFS was 10.5m vs. 7.2m (HR 0.55, (95% CI 0.26-1.17), p=0.09) in the same subgroups (Figure 5, Figure 6).

MANOVA of these clinicopathological variables potentially associated with progression free and overall survival showed that of these parameters only disease extent have a statistically significant impact on both progression free and overall survival (Table 2). Extended confounding analysis and groupwise statistical testing established that the distributions of these clinical and prognostic parameters are not different between the LOH-high and LOH-low groups (Table 3). The prognostic factors identified through MANOVA therefore confound the survival outcome of both groups equally and any additional findings can be attributed to the difference in LOH levels.

**Table 2:** Multivariate analysis of factors associated with survival in REAL3

Variable	*p* value MANOVA Overall survival	*p* value MANOVA Progression free survival
Age (<=65 (44), >65 (29))	0.83	0.64
Gender (M (7), F (67))	0.18	0.35
Tumour Site (O, S, OGJ)	0.61	0.98
Disease extent (locally advanced (7), metastatic (67))	0.04	0.01
WHO PS 0 (28), >0 (33), unknown (3)	0.23	0.25
Histological subtype (Intestinal (61), diffuse (7), Mixed (5), Unknown (1))	0.13	0.03
Treatment (EOX (38), EOX-P (36))	0.37	0.67

**Table 3:** Association between variables associated with OS and LOH

Variable	LOH-low *n*= 64	LOH-high *n*= 10	*p* value
**Age (% <65 years)**	61% (39)	50% (5)	0.87 (Gosset *t*-test)
**Gender (% F)**	8% (5)	20% (2)	1 (Fisher)
**Tumour site O, S, GOJ**	37.5% (24), 25% (16), 37.5% (24)	30% (3), 20% (2), 50% (5)	1 (Fisher)
**Disease extent (% metastatic)**	91% (58)	90% (9)	1 (Fisher)
**WHO PS (0,1,2, unknown)**	39% (25), 50% (32), 6% (4), 5% (3)	30% (3), 70% (7), 0%	1 (Fisher)
**Histological subtype (%intestinal)**	80% (51)	100% (10)	1 (Fisher)
**Treatment (EOX, EOX-P)**	53% (34), 47% (30)	40% (4), 60% (6)	1 (Fisher)

HRD can also be caused by variants in genes of the HR pathway that render the protein function impaired. Categories of variants that are deleterious to protein function include protein truncating mutations, splice site mutations, homozygous deletions and large protein truncating rearrangements. The Foundation Medicine T5 NGS assay has been validated to detect these classes of variants. In the REAL3 samples sequenced here, several deleterious mutations in genes of the HR pathway were detected and are detailed in Table 4 : *BRCA2* (n=1), *ATM* (n=6), *ATR* (n=1), *CHK2* (n=1). Additionally, 101 of 117 (86%) samples had mutations in the *TP53* gene. There was no significant difference in %LOH, OS, and PFS between the *TP53* mutated and wildtype populations, other mutant populations were considered too small to analyse separately.

**Table 4:** Overview of HR pathway mutations detected

Gene	Total number (%) samples	Number of samples with known deleterious mutation	Number of samples with mutation of unknown significance
ATM	13 (11%)	6	7
ATR	8 (7%)	1	7
ATRX	5 (4%)	2	3
BARD1	6 (5%)		6
BLM	12 (10%)		12
BRCA1	3 (3%)		3
BRCA2	13 (11%)	1	12
BRIP1	7 (6%)	1	6
CHEK1	3 (3%)		3
CHEK2	4 (3%)	1	3
FANCA	9 (8%)		9
FANCC	3 (3%)		3
FANCD2	11 (9%)		11
FANCE	6 (5%)		6
FANCF	3 (3%)		3
FANCG	1 (1%)		1
FANCI	6 (5%)		6
FANCL	4 (3%)		4
FANCM	9 (8%)		9
MRE11A	2 (2%)		2
NBN	9 (8%)		9
PALB2	6 (5%)		6
RAD50	3 (3%)		3
RAD51	1 (1%)		1
RAD51C	2 (2%)		2
RAD51D	0		0
RAD52	4 (3%)		4
RAD54L	2 (2%)		2

Abbreviations: HR, homologous recombination.

## DISCUSSION

In this analysis we identify a group of oesophagogastric cancer patients treated in the REAL3 trial who have high levels of genomic LOH measured using an algorithmic approach to DNA sequencing data performed on tissue obtained from routine diagnostic biopsies. Using a cut-off of 21% genomic LOH to define LOH-high, we demonstrate that patients with higher levels of LOH have a trend towards longer overall survival than those who are not LOH-high. When a small number of patients who underwent potentially curative surgery were censored in a sensitivity analysis, these findings reached statistical significance (HR 0.43, 95% CI (0.20-0.92), p=0.02) These findings are of interest for two reasons. Firstly, although the predictive power of the biomarker cannot be determined in the absence of a control group, high LOH may identify patients who are more likely to benefit from platinum based chemotherapy. Secondly, as LOH-high patients with ovarian cancer benefit from PARP inhibition more than LOH–low patients, it is possible that high LOH could be a biomarker predictive of sensitivity to PARP inhibitors in oesophagogastric cancer patients. While these hypotheses require prospective validation, they could lead to more effective biomarker selected therapies for oesophagogastric cancer, a disease which currently has a dismal prognosis [18, 19].

Platinum based chemotherapy is a standard first line treatment for patients with advanced oesophagogastric cancer [20–23]. To date, no biomarker is available to select which oesophagogastric cancer patients might benefit from chemotherapy. Many studies have evaluated germline polymorphisms associated with response to platinum and fluoropyrimidine chemotherapy, but none have demonstrated definitive or practice changing results [24–26]. Microsatellite instability may be a biomarker of chemosensitivity for gastric cancer patients treated with perioperative chemotherapy; however this is rare in proximal tumours and in the the metastatic setting [27, 15]. Therefore, with the exception of HER2 overexpression and trastuzumab treatment, useful biomarkers to stratify patients with oesophagogastric cancer for standard therapy are lacking [28]. We suggest further evaluation of the LOH-high biomarker in clinical trials in patients with oesophagogastric cancer in order to validate our findings.

The genomic and transcriptomic landscape of oesophagogastric cancer has been intensively explored recently. The recently published oesophageal TCGA data suggests that oesophageal adenocarcinoma is molecularly almost indistinguishable from chromosomally unstable gastric cancer [15]. These cancers are characterized by gross genomic instability and frequent large scale chromosomal events such as kataegis and chromothripsis which can lead to defects in homologous recombination and acquisition of genomic scar [29]. Gastric cancer has previously been identified as having a comparatively high level of genomic scarring measured using not only LOH, but also quantified using other metrics such as telomeric allelic imbalances (NtAI) and large scale transitions (LST), both of which predict platinum sensitivity in triple negative breast cancer [10, 30, 31]. Thus, although the presence of the HRD tumour phenotype and its association with platinum sensitivity has been established by multiple different methodologies across several cancer types including gastric cancer, we are the first to demonstrate potentially better survival with the HRD phenotype in oesophagogastric cancer treated with platinum chemotherapy and do so in a well annotated phase III randomized trial population. Based on patterns of presentation, it is likely that most of our samples were collected from the primary tumour, however as this information was not collected systematically, we cannot comment on the relationship between LOH, primary tumours and metastases.

Recent seminal work has demonstrated that chromosomal instability drives metastasis independently of aneuploidy and has described increased levels chromosomal instability in a cohort of matched primary tumour and brain metastases, in addition to a series of matched breast cancer primaries and metastases [32]. Therefore it is possible that our work, which was conducted on primary tumours, could underestimate the level of LOH in gastroesophageal cancer metastasis. This also in turn provides a potential explanation for divergent responses of primary and metastatic sites to platinum chemotherapy.

PARP inhibitors have a well-defined role in ovarian cancer, the value of PARP inhibition in oesophagogastric cancer is less clear. The Phase III randomized phase III GOLD trial failed to demonstrate a statistically significant overall survival advantage for olaparib treated patients in intention to treat trial population (median OS 8.8 months vs. 6.9 months (HR = 0.79, P =.0262), although this may be in part due to a statistical correction for multiple primary endpoints [33]. Notably, patients who were ATM negative and treated with olaparib demonstrated substantially improved response rates compared to ATM negative patients treated with paclitaxel alone (ORR 4.24, p=0.0309), therefore PARP inhibitors may still be effective in the correct biomarker selected oesophagogastric cancer population [34]. Our biomarker may identify a complementary group of gastroesophageal cancer patients who are ATM positive, yet who could benefit from a DNA damage targeting therapeutic approach. The potential value of a DNA damaging targeting approach in oesophageal cancers is also demonstrated by a recent large whole genome sequencing study of oesophageal adenocarcinoma which identified three intrinsic genomic signatures, one of which was putatively sensitive to PARP inhibition; however functional and clinical validation of this finding is awaited [16].

One limitation of our study is the small proportion of tumours from the total trial population which were successfully sequenced; prior biomarker studies on the same population had exhausted much of the available tissue [35]. In particular, the modest number of patients included in survival analyses and the imbalance between LOH-high and low-groups could hinder comparisons between the groups and inclusion of LOH status into the multivariate model. However, as there was no significant difference between the survival of patients included in this study and the original trial, we do not think this introduced significant bias. We also did not find any confounding effects from other clinicopathological variables. Finally, a further limitation of our work is the lack of a control group, as all patients in our study received platinum chemotherapy; the predictive versus prognostic value of the LOH signature will need to be evaluated in further research. We think that there is sufficient indirect evidence to support our hypothesis, which is that of genomic LOH (a measure of HRD), could be associated with clinical benefit from platinum based chemotherapy in oesophagogastric cancer.

In conclusion, in this study we present our results from deriving a genomic signature for high LOH in oesophagogastric cancer patients treated with platinum based chemotherapy in the REAL3 trial, which we found to be prognostic for survival in this patient population. The results of the current study can be considered preliminary, and analysis of a larger cohort is necessary in order to provide further validation on the hypothesized clinical significance of LOH. Specifically, we acknowledge that the small size of our data set made it impossible to separate the data into a training data set and one to test the LOH cutoff prospectively. We plan to do this in the phase II randomized PLATFORM trial (NCT02678182) which is currently running in the United Kingdom and which will have recruited almost one thousand patients when fully accrued. As immuno-oncology therapy moves to the fore, tumours with high levels of genomic scar which elicit robust immune responses may also be candidates for immune checkpoint therapy, and it is possible that combining PARP inhibition and checkpoint inhibitor therapy could provide long term benefits for selected patients [36, 37].

## MATERIALS AND METHODS

From the REAL3 cohort (n=553) pre-treatment tumour biopsies (tissue blocks) with high tumour content (>30%) were selected by a pathologist. All patients included in this analysis had given informed consent for translational research. The REAL3 trial was conducted under national and local ethical approvals.

The Foundation Medicine T5 next-generation sequencing assay (Foundation Medicine, Cambridge, MA, USA) was used to calculate the percentage of genomic LOH in pretreatment biopsies, a minimum DNA input of 200ng is recommended for the assay [38]. This assay interrogates 287 cancer-related genes for mutations and 3543 single-nucleotide polymorphisms (SNPs) across the whole genome. An algorithm was developed to quantify the percentage of interrogable genome with LOH. Briefly, minor-allele frequencies of the examined SNPs and copy number profile across the 22 autosomal chromosomes were used to identify segments with LOH across the interrogable genome. Excluded from this percentage were events that were unlikely to be caused by HRD mechanisms, such as whole chromosome or chromosome-arm loss. The percentage of genomic LOH for each sample was calculated as the sum of the lengths of included LOH segments divided by the length of the interrogated genome.

The primary endpoint of the study was to determine a cut-off for LOH which separated patients into two groups (LOH-high and LOH-low) which were associated with distinct survival outcomes. To set a cutoff for separating samples high in genomic LOH from those low in genomic LOH, we performed a multi-dimensional optimization of parameters across all possible LOH cutoffs across the range of observed LOH values [39]. Across the range of genomic LOH 3% to 26% and in increments of 1% we calculated the following values; hazard ratio (HR) between LOH high and low populations using a Cox proportional hazards regression model; the likelihood ratio p-value of said hazard ratio; median overall and progression free survival (OS, PFS) in the LOH high and low groups and the size of the LOH-high vs. low populations captured by that cutoff. We selected the smallest HR with the smallest p-value and required sensitivity and specificity to be larger than 50% for both OS and PFS. From the subset of LOH cutoffs that meet these criteria, we chose the one that captured the largest patient population within these criteria.

Multivariate analysis of variance (MANOVA) was used to identify variables that could potentially confound any findings related to survival. The investigated potentially confounding variables were: age, gender, tumour site, disease extent, WHO performance status, histological subtype, and treatment group. To control for the effects of potentially confounding variables we performed an extended confounding analysis and applied groupwise statistical tests to the parameters above in the two groups, LOH-high and LOH-low. In order to homogenise the patient population we performed a sensitivity analysis in which patients who underwent potentially curative surgery after chemotherapy were censored for progression free and overall survival at the time of surgery.

## SUPPLEMENTARY MATERIALS FIGURE



## Abbreviations

HRD: homologous recombination deficiency
LOH: loss of heterozygosity
MANOVA: multivariate analysis of variance
OS: overall survivall
PFS: progression free survival
USA: United States of America.

## References

[1] Pearl LH, Schierz AC, Ward SE, Al-Lazikani B, Pearl FM (2015). Therapeutic opportunities within the DNA damage response. Nat Rev Cancer.

[2] Moynahan ME, Pierce AJ, Jasin M (2001). BRCA2 is required for homology-directed repair of chromosomal breaks. Mol Cell.

[3] Moynahan ME, Chiu JW, Koller BH, Jasin M (1999). Brca1 controls homology-directed DNA repair. Mol Cell.

[4] Ledermann J, Harter P, Gourley C, Friedlander M, Vergote I, Rustin G, Scott CL, Meier W, Shapira-Frommer R, Safra T, Matei D, Fielding A, Spencer S (2014). Olaparib maintenance therapy in patients with platinum-sensitive relapsed serous ovarian cancer: a preplanned retrospective analysis of outcomes by BRCA status in a randomised phase 2 trial. Lancet Oncol.

[5] Gelmon KA, Tischkowitz M, Mackay H, Swenerton K, Robidoux A, Tonkin K, Hirte H, Huntsman D, Clemons M, Gilks B, Yerushalmi R, Macpherson E, Carmichael J (2011). Olaparib in patients with recurrent high-grade serous or poorly differentiated ovarian carcinoma or triple-negative breast cancer: a phase 2, multicentre, open-label, non-randomised study. Lancet Oncol.

[6] Mateo J, Carreira S, Sandhu S, Miranda S, Mossop H, Perez-Lopez R, Nava Rodrigues D, Robinson D, Omlin A, Tunariu N, Boysen G, Porta N, Flohr P (2015). DNA-Repair Defects and Olaparib in Metastatic Prostate Cancer. The New England Journal of Medicine.

[7] Swisher EM, Lin KK, Oza AM, Scott CL, Giordano H, Sun J, Konecny GE, Coleman RL, Tinker AV, O'Malley DM, Kristeleit RS, Ma L, Bell-McGuinn KM (2017). Rucaparib in relapsed, platinum-sensitive high-grade ovarian carcinoma (ARIEL2 Part 1): an international, multicentre, open-label, phase 2 trial. Lancet Oncol.

[8] Watkins JA, Irshad S, Grigoriadis A, Tutt AN (2014). Genomic scars as biomarkers of homologous recombination deficiency and drug response in breast and ovarian cancers. Breast Cancer Res.

[9] Telli ML, Timms KM, Reid J, Hennessy B, Mills GB, Jensen KC, Szallasi Z, Barry WT, Winer EP, Tung NM, Isakoff SJ, Ryan PD, Greene-Colozzi A (2016). Homologous Recombination Deficiency (HRD) Score Predicts Response to Platinum-Containing Neoadjuvant Chemotherapy in Patients with Triple-Negative Breast Cancer. Clin Cancer Res.

[10] Marquard AM, Eklund AC, Joshi T, Krzystanek M, Favero F, Wang ZC, Richardson AL, Silver DP, Szallasi Z, Birkbak NJ (2015). Pan-cancer analysis of genomic scar signatures associated with homologous recombination deficiency suggests novel indications for existing cancer drugs. Biomark Res.

[11] Lin K, Sun J, Maloney L, Goble S, Oza A, Coleman R, Scott C, Robillard L, Mann E, Isaacson J (2015). 2701 quantification of genomic loss of heterozygosity enables prospective selection of ovarian cancer patients who may derive benefit from the PARP inhibitor rucaparib. European Journal of Cancer.

[12] Bang YJ, Im SA, Lee KW, Cho JY, Song EK, Lee KH, Kim YH, Park JO, Chun HG, Zang DY, Fielding A, Rowbottom J, Hodgson D (2015). Randomized, Double-Blind Phase II Trial With Prospective Classification by ATM Protein Level to Evaluate the Efficacy and Tolerability of Olaparib Plus Paclitaxel in Patients With Recurrent or Metastatic Gastric Cancer. J Clin Oncol.

[13] Kubota E, Williamson CT, Ye R, Elegbede A, Petersen L, Lees-Miller SP, Bebb DG (2014). Low ATM protein expression and depletion of p53 correlates with olaparib sensitivity in gastric cancer cell lines. Cell Cycle.

[14] The Cancer Genome Atlas Research Network (2014). Comprehensive molecular characterization of gastric adenocarcinoma. Nature.

[15] The Cancer Genome Atlas Research Network (2017). Integrated genomic characterization of oesophageal carcinoma. Nature.

[16] Secrier M, Li X, de Silva N, Eldridge MD, Contino G, Bornschein J, MacRae S, Grehan N, O'Donovan M, Miremadi A, Yang TP, Bower L, Chettouh H, Oesophageal Cancer Clinical and Molecular Stratification (OCCAMS) Consortium (2016). Mutational signatures in esophageal adenocarcinoma define etiologically distinct subgroups with therapeutic relevance. Nat Genet.

[17] Waddell T, Chau I, Cunningham D, Gonzalez D, Okines AF, Okines C, Wotherspoon A, Saffery C, Middleton G, Wadsley J, Ferry D, Mansoor W, Crosby T (2013). Epirubicin, oxaliplatin, and capecitabine with or without panitumumab for patients with previously untreated advanced oesophagogastric cancer (REAL3): a randomised, open-label phase 3 trial. Lancet Oncol.

[18] Siegel RL, Miller KD, Jemal A (2016). Cancer statistics, 2016. CA Cancer J Clin.

[19] Cunningham D, Starling N, Rao S, Iveson T, Nicolson M, Coxon F, Middleton G, Daniel F, Oates J, Norman AR, Upper Gastrointestinal Clinical Studies Group of the National Cancer Research Institute of the United Kingdom (2008). Capecitabine and Oxaliplatin for Advanced Esophagogastric Cancer. The New England Journal of Medicine.

[20] Smyth EC, Verheij M, Allum W, Cunningham D, Cervantes A, Arnold D, ESMO Guidelines Committee (2016). Gastric cancer: ESMO Clinical Practice Guidelines for diagnosis, treatment and follow-up. Ann Oncol.

[21] Lordick F, Mariette C, Haustermans K, Obermannova R, Arnold D (2016). Oesophageal cancer: ESMO Clinical Practice Guidelines for diagnosis, treatment and follow-up. Ann Oncol.

[22] National Comprehensive Cancer Network (2016). NCCN Esophageal Cancer Guidelines.

[23] National Comprehensive Cancer Network (2015). NCCN Cancer Guidelines.

[24] Goekkurt E, Al-Batran SE, Hartmann JT, Mogck U, Schuch G, Kramer M, Jaeger E, Bokemeyer C, Ehninger G, Stoehlmacher J (2009). Pharmacogenetic analyses of a phase III trial in metastatic gastroesophagealadenocarcinoma with fluorouracil and leucovorin plus either oxaliplatin or cisplatin: a study of the arbeitsgemeinschaft internistische onkologie. Journal of Clinical Oncology.

[25] Ruzzo A, Graziano F, Kawakami K, Watanabe G, Santini D, Catalano V, Bisonni R, Canestrari E, Ficarelli R, Menichetti ET, Mari D, Testa E, Silva R (2006). Pharmacogenetic profiling and clinical outcome of patients with advanced gastric cancer treated with palliative chemotherapy. Journal of Clinical Oncology.

[26] Patel JN, Fuchs CS, Owzar K, Chen Z, McLeod HL (2013). Gastric cancer pharmacogenetics: progress or old tripe?. Pharmacogenomics.

[27] Smyth EC, Wotherspoon A, Peckitt C, Gonzalez D, Hulkki-Wilson S, Eltahir Z, Fassan M, Rugge M, Valeri N, Okines A, Hewish M, Allum W, Stenning S (2017). Mismatch Repair Deficiency, Microsatellite Instability, and Survival : An Exploratory Analysis of the Medical Research Council Adjuvant Gastric Infusional Chemotherapy (MAGIC) Trial. JAMA Oncol.

[28] Bang YJ, Van Cutsem E, Feyereislova A, Chung HC, Shen L, Sawaki A, Lordick F, Ohtsu A, Omuro Y, Satoh T, Aprile G, Kulikov E, Hill J, ToGA Trial Investigators (2010). Trastuzumab in combination with chemotherapy versus chemotherapy alone for treatment of HER2-positive advanced gastric or gastro-oesophageal junction cancer (ToGA): a phase 3, open-label, randomised controlled trial. The Lancet.

[29] Nones K, Waddell N, Wayte N, Patch AM, Bailey P, Newell F, Holmes O, Fink JL, Quinn MC, Tang YH, Lampe G, Quek K, Loffler KA (2014). Genomic catastrophes frequently arise in esophageal adenocarcinoma and drive tumorigenesis. Nat Commun.

[30] Birkbak NJ, Wang ZC, Kim JY, Eklund AC, Li Q, Tian R, Bowman-Colin C, Li Y, Greene-Colozzi A, Iglehart JD, Tung N, Ryan PD, Garber JE (2012). Telomeric allelic imbalance indicates defective DNA repair and sensitivity to DNA-damaging agents. Cancer Discov.

[31] Popova T, Manie E, Rieunier G, Caux-Moncoutier V, Tirapo C, Dubois T, Delattre O, Sigal-Zafrani B, Bollet M, Longy M, Houdayer C, Sastre-Garau X, Vincent-Salomon A (2012). Ploidy and large-scale genomic instability consistently identify basal-like breast carcinomas with BRCA1/2 inactivation. Cancer Res.

[32] Bakhoum SF, Ngo B, Laughney AM, Cavallo JA, Murphy CJ, Ly P, Shah P, Sriram RK, Watkins TBK, Taunk NK, Duran M, Pauli C, Shaw C (2018). Chromosomal instability drives metastasis through a cytosolic DNA response. Nature.

[33] Bang YJ, Xu RH, Chin K, Lee KW, Park SH, Rha SY, Shen L, Qin S, Xu N, Im SA, Locker G, Rowe P, Shi X (2017). Olaparib in combination with paclitaxel in patients with advanced gastric cancer who have progressed following first-line therapy (GOLD): a double-blind, randomised Phase III trial. Lancet Oncol.

[34] Smyth E (2017). Missing a GOLDen opportunity in gastric cancer. Lancet Oncol.

[35] Okines AF, Gonzalez de Castro D, Cunningham D, Chau I, Langley RE, Thompson LC, Stenning SP, Saffery C, Barbachano Y, Coxon F, Middleton G, Ferry D, Crosby T (2013). Biomarker analysis in oesophagogastric cancer: Results from the REAL3 and TransMAGIC trials. Eur J Cancer.

[36] McAlpine JN, Porter H, Kobel M, Nelson BH, Prentice LM, Kalloger SE, Senz J, Milne K, Ding J, Shah SP, Huntsman DG, Gilks CB (2012). BRCA1 and BRCA2 mutations correlate with TP53 abnormalities and presence of immune cell infiltrates in ovarian high-grade serous carcinoma. Mod Pathol.

[37] Jiao S, Xia W, Yamaguchi H, Wei Y, Chen MK, Hsu JM, Hsu JL, Yu WH, Du Y, Lee HH, Li CW, Chou CK, Lim SO (2017). PARP Inhibitor Upregulates PD-L1 Expression and Enhances Cancer-Associated Immunosuppression. Clin Cancer Res.

[38] Frampton GM, Fichtenholtz A, Otto GA, Wang K, Downing SR, He J, Schnall-Levin M, White J, Sanford EM, An P, Sun J, Juhn F, Brennan K (2013). Development and validation of a clinical cancer genomic profiling test based on massively parallel DNA sequencing. Nat Biotechnol.

[39] Mazumdar M, Glassman JR (2000). Categorizing a prognostic variable: review of methods, code for easy implementation and applications to decision-making about cancer treatments. Stat Med.

