# Supplementary material for: Genomic loss of heterozygosity and survival in the REAL3 trial
Source: Oncotarget. 2018 Nov 30;9(94):36654–65. doi: 10.18632/oncotarget.26336 (PMC6291175; doi:10.18632/oncotarget.26336)
Supplement: Supplementary file 1 [file oncotarget-09-36654-s001.pdf]

Genomic loss of heterozygosity and survival in the REAL3 trial

SUPPLEMENTARY MATERIALS

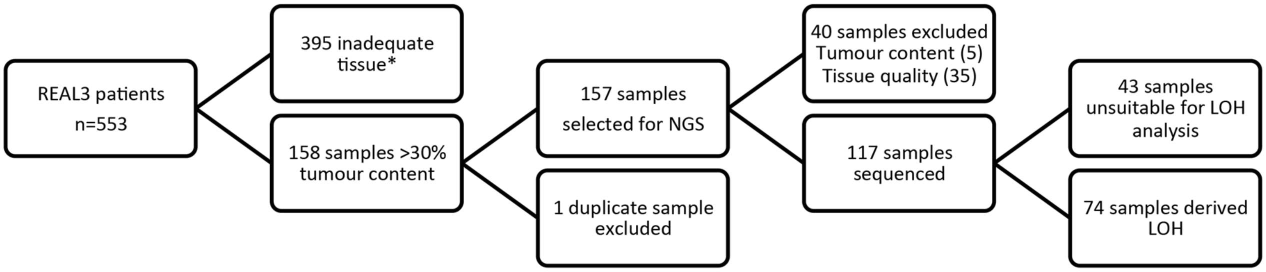

Supplementary Data: REAL3 LOH REMARK Diagram. LOH, loss of heterozygosity; NGS, next generation sequencing. *Samples used for prior translational studies.
